# Exposure to fine particulate matter in the New York City subway system during home-work commute

**DOI:** 10.1371/journal.pone.0307096

**Published:** 2024-08-07

**Authors:** Shams Azad, Pau Ferrer-Cid, Masoud Ghandehari

**Affiliations:** 1 Department of Civil and Urban Engineering, New York University, Tandon School of Engineering, Brooklyn, New York, United States of America; 2 Lamont-Doherty Earth Observatory, Columbia Climate School, Columbia University, New York, New York, United States of America; 3 Department of Computer Architecture, Universitat Politècnica de Catalunya, Barcelona, Spain; Chang’an University, CHINA

## Abstract

The New York City (NYC) subway system accommodates 5.5 million daily commuters, and the environment within the subway is known to have high concentrations of fine particulate matter (PM_2.5_) pollution. Naturally, subway air pollution varies among individuals according to their mobility patterns, introducing the possibility of inequality in PM_2.5_ exposure. This study aims to evaluate individual and community-level exposure to subway PM_2.5_. We simulated the intracity home-to-work trip patterns using the Longitudinal Employer-Household Dynamics (LEHD) records of 3.1 million working commuters across 34,169 census blocks in four boroughs (Manhattan, Brooklyn, Queens, and the Bronx) of NYC. We incorporated the on-platform and on-train measured PM_2.5_ concentration data for the entire subway system. The mean underground platform concentration in the city was 139 μg/m^3^ with a standard deviation of 25 μg/m^3^, while the on-train concentration when underground was 99 μg/m^3^ with a standard deviation of 21 μg/m^3^. Using a network model, we determined the exposure of individual commuters during their daily home-work trips. We quantified the mean per capita exposure at the census block level by considering the proportion of workers within the blocks who rely on the subway for their work commute. Results indicate statistically significant weak positive correlation between elevated subway PM_2.5_ exposure and economically disadvantaged and racial minority groups.

## Introduction

Particulate matter (PM) is a complex mixture of solid and liquid particles comprising a range of inorganic and organic chemicals [1]. PM_2.5_ refers to the PM with an aerodynamic diameter equal to or smaller than 2.5 μm [2]. Due to their small size, these particles can remain suspended in the air for long periods, and when inhaled, they can easily enter the bloodstream. This can cause short-term and long-term health complications, including cardiovascular, respiratory, metabolic, and neurological disorders [3–13]. Approximately 4.1 million people prematurely die worldwide each year due to exposure to PM_2.5_ [14].

For the last few decades, cities worldwide have promoted public transportation systems to reduce traffic congestion and improve air quality. These measures have helped reduce emissions and improve city ambient outdoor air quality [15–18]. Although subway systems effectively reduce ambient air pollution by decreasing the number of fossil fuel-powered vehicles on the road, the air quality inside the subway system is poor. This is largely due to elevated concentrations of PM_2.5_ with high concentrations of iron [19–28]. Metal-rich particles in subway systems are mostly generated by the wear and friction of brakes and between rails and wheels [29]. Contributing factors for the high PM_2.5_ concentrations in subways include train frequency, station depth, ventilation, age of the subway system, piston effect, and others. [30–33].

When assessing health impacts, the total exposure and the inhaled dose are commonly used as representative measures of exposure to pollutants such as PM_2.5_ in indoor and outdoor settings [34–42]. Several studies have been carried out on personal exposure in environments such as the subway and bus systems of a city, using the inhaled dose of PM_2.5_ [34, 37, 43]. The total exposure and the inhaled dose depend on the mean inhalation rate during a trip, the mean concentration during a trip, and the trip duration [35, 36, 38]. In complex cases, exposure has been calculated as the sum of exposures in different microenvironments, such as inside a subway train, waiting on a platform, or walking [38]. To evaluate the exposure city residents face in transportation systems, network-based models are generally used to simulate mobility patterns between different parts of a city, following the most common requirement and the fastest route to estimate the air pollution to which commuters have been exposed when they have traveled using that route [41, 42, 44, 45]. For instance, [41] calculated the shortest routes for different locations to simulate mobility patterns and compute the total exposure associated with those trips.

Existing research on subway PM_2.5_ has focused on a variety of topics, including measuring air pollution concentrations in subway systems [24, 46–54], identifying the factors that contribute to air pollution and mitigation strategies [55–58], and assessing the health effects of subway PM_2.5_ exposure [59–63]. However, no studies to date have investigated inequality in subway PM_2.5_ exposure at the community level while accounting for daily intracity subway mobility. It is important to note that not everyone is equally exposed to subway air pollution, even if the system as a whole is polluted. Individual exposure is influenced by the frequency and duration of subway use, which introduces the potential for inequality in subway-related exposure. As a result, we hypothesize that economically disadvantaged communities and racial minority groups living farther from the city center may take more frequent and longer subway trips, thus disproportionately exposed to subway PM_2.5_. The main objective of this study is to assess individual and community level PM_2.5_ exposure and uncover any disparities in subway-related exposure. To achieve this goal, we carry out our analysis in three distinct phases. In the first phase, we focus on measuring PM_2.5_ concentrations across the entire subway system. This phase builds upon our prior investigation [26], which examined PM_2.5_ concentrations on subway trains and platforms within nine subway lines. In this study, we expanded our research to include the assessment of particle concentrations on an additional ten subway lines, resulting in a comprehensive analysis of on-train concentrations for a total of 19 subway lines and on-platform concentrations for 608 subway station platforms located within 429 stations. The second phase involves quantifying PM_2.5_ exposure associated with subway usage at the individual and community levels. To accomplish this, we utilized LEHD origin-destination (OD) data [64] to simulate the daily commutes of 3.1 million working individuals on 34,169 census blocks in New York City. We constructed a network model to analyze mobility patterns and calculate the corresponding subway PM_2.5_ exposure for each origin and destination. In the third phase, we conducted correlative analyses to highlight any disparities in exposure resulting from the use of the subway system in NYC.

## 2. Materials and methods

### 2.1 Study region

New York City comprises five boroughs: Manhattan, Brooklyn, Queens, the Bronx, and Staten Island. Staten Island has a standalone subway line not connected to the rest of the city’s subway network. This study focused on the four boroughs with the connected subway system. The working population within these four boroughs is approximately 3.6 million, among which 3.1 million live and work in the city, while the remaining 0.5 million commute outside the city for work [64]. Our study focuses specifically on the mobility of work-home trips for these 3.1 million workers who reside and work within the study area.

**Fig 1:**
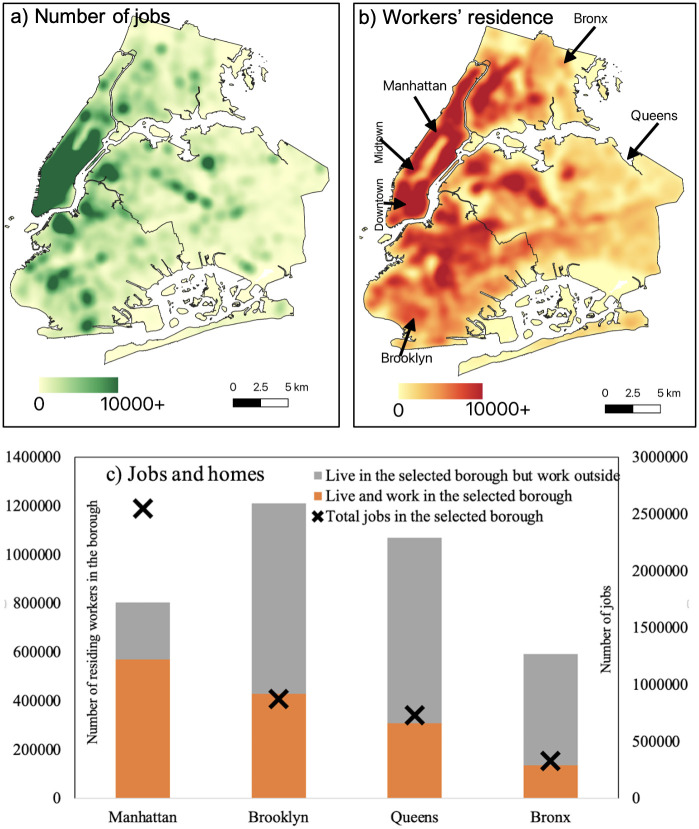
Home-job mobility dynamics. (a) Heatmap showing the number of jobs, (b) Shows a heatmap of where workers live. The kernel density estimation is done with 1-km bandwidth and 100-meter pixel size. (c) Displays the distribution of workers’ residential and workplace locations at the borough level, along with the number of jobs in each borough.

Fig 1(a) shows the distribution of jobs across the city. Manhattan accommodates over 2.5 million jobs within its boundaries. Most jobs in this borough are concentrated in downtown and midtown. Approximately 71% of workers who live in Manhattan also work within Manhattan. However, Brooklyn, Queens, and the Bronx workers often have to commute to other boroughs for employment. Only 35% of workers in Brooklyn, 29% in Queens, and 22% in the Bronx work within the same borough in which they reside. Fig 1(b) shows the distribution of workers across the city based on their residence. Lastly, Fig 1(c) presents a detailed representation of the home-job dynamics at the borough level, categorizing workers into three distinct groups: those who live and work within the same borough, those who live in one borough and commute to work in another borough, and the total number of jobs available in each borough.

### 2.2 Workers’ intracity mobility

We used the 2019 LEHD Origin-Destination (OD) dataset [64] to map the mobility of workers traveling from their homes to their places of work within the city. The LEHD dataset provides OD data at the census block level. This data also provides information on the number of workers residing in each block and their workplaces. According to the 2019 census block boundary [65], there are 34,169 blocks in our study area. Of these, 25,694 census blocks have at least one worker in the city. On average, 119 workers live in each block, with a standard deviation of 135. We have used data for 2019 because, in our analysis, we wanted to exclude the changes in mobility patterns following the onset of the COVID-19 pandemic in 2020 [66, 67]

**Fig 2:**
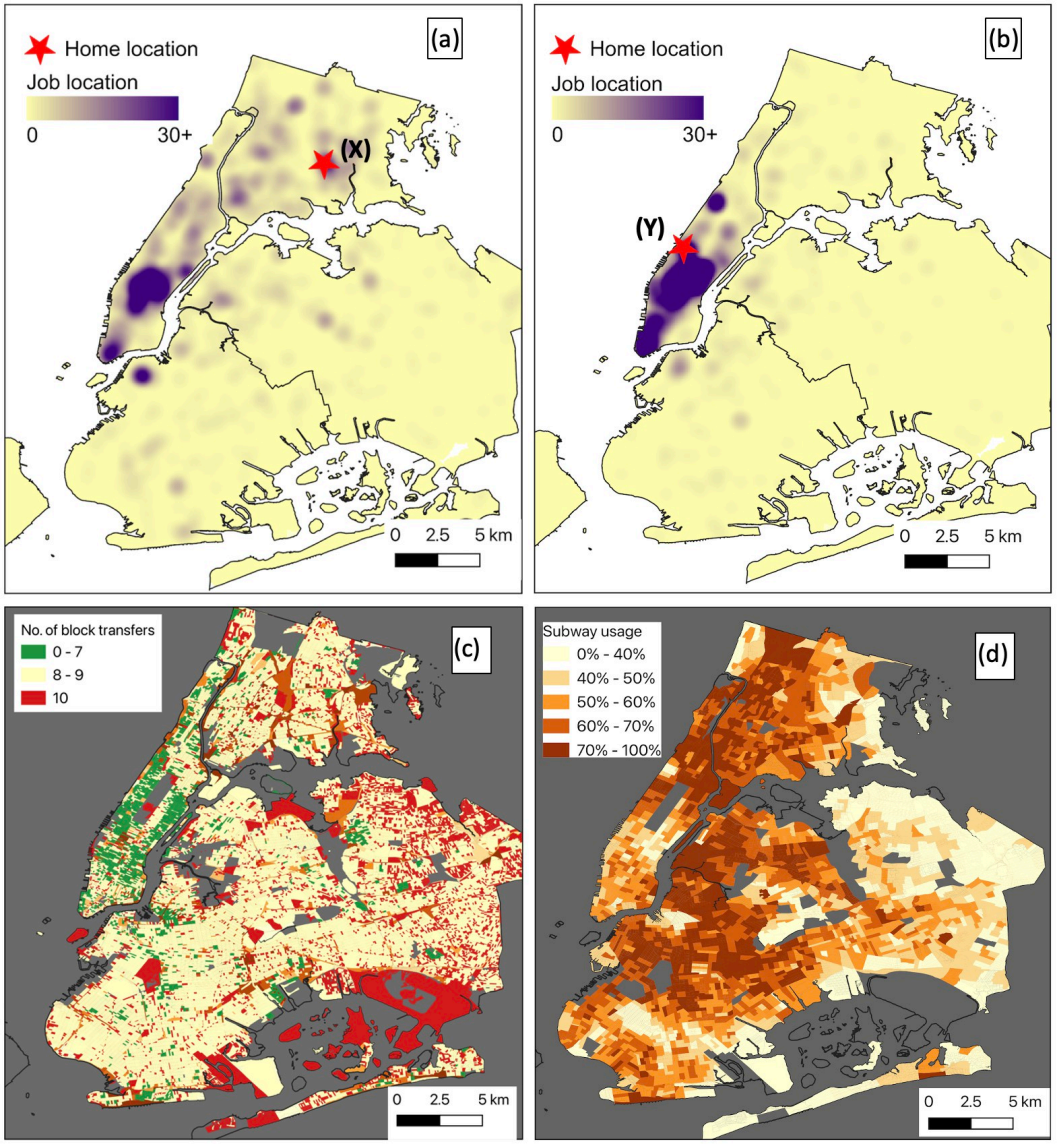
Home-job trips at the census block level. (a) Heatmap Home to job mobility for block ‘X’ located in the Bronx, (b) Heatmap Home to job mobility for block ‘Y’ located in Manhattan, Kernel density estimation is done with 1-km bandwidth and 100-meter pixel size. (c) Number of destinations blocks from each origin block for 10 workers. (d) Percent of workers using public transportation for work.

Workers living in one block often need to travel to other blocks for work. Those who live further away from their job take longer subway rides to get to work, increasing their exposure to high subway PM_2.5_. Fig 2(a) and 2(b) shows the home-to-job mobility patterns of two examples (Block X Fig 2a and YFig 2b). The red star represents the origin block, with block ‘X’ in the Bronx accommodating 965 workers and block ‘Y’ in midtown Manhattan accommodating 732 workers. The heatmap in the figure shows where these workers go to work. For block ‘X’, where there are not enough jobs around, the heatmap indicates that most workers living in block ‘X’ travel to midtown Manhattan, where most jobs are concentrated. Some even travel to downtown Manhattan and Brooklyn, with very few working in the Bronx, which is their home borough. In contrast, block ‘Y’ is located in midtown Manhattan, and the workers who live in this block work in midtown and downtown Manhattan, which is very close to their residence. Unlike block ‘X’ workers, block ‘Y’ workers do not need to travel far. As a result, they are less vulnerable to subway exposure. We have made a web platform with this LEHD OD data which can be utilized to investigate the workers’ mobility at the census block level. This platform can be accessed through this link: https://bit.ly/3MK7B4r.

When a census block has a significant number of job opportunities, many workers who live there may not need to travel to other blocks for work. In such cases, some workers live and work in the same block, which can significantly reduce the necessity of subway travel for work. Fig 2(c) displays the mobility patterns of workers, which are determined by the number of blocks they travel to for work, measured at a ratio of ten workers per block. The green color-coded blocks in the figure indicate that, for every ten workers, at least three workers work within the same block they live in, reducing the need to travel to other blocks. In contrast, the red shaded blocks show that all ten workers need to travel to other blocks to work, increasing the need for travel and potential exposure to subway air pollution.

ACS provides data on the proportion of workers who use public transit for their commute at the census tract level (2057 census tracts in the study area); this includes subway and public buses [68]. While we do not have the exact number of subway users at census block level, we have approximated that the majority of workers who use public buses likely use them to get to the closest subway station, and therefore, most of them eventually use the subway. We disaggregated the census tract-level transit usage data to the census block level.

Fig 2(d) illustrates the percentage of workers who use the subway for their home-to-job commute. The map shows that workers living in midtown and downtown Manhattan use the subway less frequently compared to those residing in other areas. This indicates the people who live in these areas may use private transportation or carpool services or live in close proximity to their workplace. However, subway usage is also low in the outskirts of Queens. This is likely due to the lack of nearby subway stations in those places, which forces workers to rely on private transportation to get to work.

### 2.3 Measurement of PM_2.5_ concentration in the subway system

This study used sampling data from our previous study [26], which offered information on PM_2.5_ concentration on subway station platforms and inside train cabins for nine subway lines. However, to incorporate the entire NYC subway system, this study conducted additional measurements of PM_2.5_ concentration for stations and train cabins, which were not covered by the previous study.

We measured PM_2.5_ concentration on the station platforms and inside train cabins by taking a round trip of each subway line. First, we measured on-train concentration by co-locating real-time and gravimetric instruments while the train moved from the first station to the last station on its route. On the return journey, we got off at each station along the line and collected samples of air pollution concentrations on the platform until the next train arrived on the same line. We spent around 5–15 min on each platform before boarding the train to the next station. For real-time PM_2.5_ measurements, we utilized Nephelometric-based DataRAM pDR 1500 units (pDR) manufactured by Thermo Fisher Scientific Inc. PDRs were equipped with a 2.5 μm diameter cut point inlet cyclone and underwent calibration with gravimetric PM_2.5_ concentrations. The pDRs collected real-time measurements at 1-s intervals and were zeroed with HEPA-filtered air before the start of each sampling run. The mean concentration for each platform is determined by calculating the mean value of measurements taken at 1-s intervals over a duration of 5 to 15 min, which depends on how long the investigators remained on the platform for sampling. We also report the on-train concentration for each link, which represents the segment between two stations. This is calculated by determining the mean concentration inside the train car from the moment the doors close at one station until they open at the next station. During sampling, we recorded the times of door opening and closing at stations, as well as boarding and disembarking times for each station. During post-processing, we filtered the real-time data using these recorded times to calculate the mean on-platform concentration in each station and on-train concentrations for each link. We used MTA defined publicly available subway station and route data to visualize the concentration data on maps. The on-train and on-platform concentration for the NYC subway system is shown in Fig 4.

### 2.4 Network model

In order to evaluate the exposure, it is necessary to profile the mobility patterns from one block to another. Therefore, it is reasonable to assume that commuters use the fastest route to get from one subway station to another, so a model of the NYC subway system is needed to calculate the shortest paths [69]. Therefore, in order to estimate mobility patterns, the shortest paths between city blocks can be calculated [41, 42, 44]. A very common approach in the modeling of transport networks consists of modeling a transport system by means of a network [36, 45, 70–72]. A network *G* = {*V*, *E*, ***W***} can be used to represent a subway system, where *V* = {*v*_1_,…, *v*_*N*_} is the set of graph nodes, where *N* is the number of network nodes. *E* = {(*v*_*i*_, *v*_*j*_): *v*_*i*_, *v*_*j*_ ∈ *V*} is the set of edges, represented by a set of vertex tuples, where *e*_*ij*_ ∈ *E* represents an edge connecting nodes *v*_*i*_ and *v*_*j*_, i.e., a path between two stations. Finally, $W\in R^{NxN}$ is the matrix of costs, where *W*_*ij*_ is the cost associated to edge *e*_*ij*_. There are different variants that can be used depending on the network, in the case of the subway, it makes sense to use an undirected graph, where the existence of a subway line in one direction implies the existence of the line in the opposite direction, i.e., *e*_*ij*_ implies the existence of *e*_*ji*_. Moreover, there is an additional challenge with subway networks, which is the existence of multiple lines between different stations as well as the possibility of commuting from one line to another and transferring from one subway station to another.

**Fig 3:**
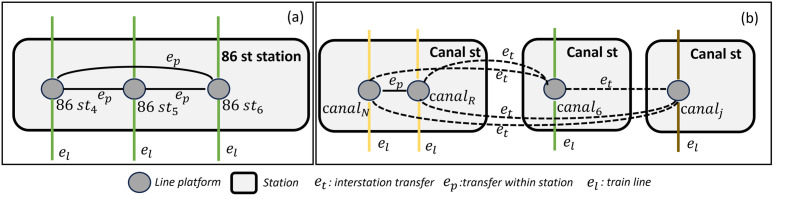
Network model architecture. (a) Stations are represented by their corresponding line platforms, e.g., the 86^th^ Street Station has three different lines, and edges e_p_ mark the cost of transferring from one line to another within the same station. (b) Shows four different line platforms from different stations that are reachable by a transfer, denoted by the edges e_t_. As illustrated, there are three different stations in Canal Street where interstation transfers can be made.

In this study, we create a network *G* where the nodes represent line platforms within the different stations, i.e., each node represents the specific platform of subway station’s line. The set of edges *E* is formed by edges connecting stations along the same line *E*_*l*_, edges connecting line platforms within the same station *E*_*p*_, and edges connecting line platforms from different stations where there is the possibility of transferring *E*_*t*_, so *E* = *E*_*l*_ ∪ *E*_*p*_ ∪ *E*_*t*_. We have decided to use this network approach to penalize possible line changes within the same subway station. Fig 3 shows an example of how different stations are represented by the network as well as the different station transfers and line transfers.

Finally, costs *W*_*ij*_ must be assigned to the different connections between nodes, so as to reflect the cost of going from one station to another. Usually, the most important metric for commuters is time, so we can define the cost *W*_*ij*_ as follows,

$$W_{ij}=\left\{\begin{array}{c} \frac{d_{ij}}{v_{avg}}\;,\;e_{ij}\in E_{l}\\ t_{p}\;,\;e_{ij}\in E_{p}\\ t_{t}\;,\;e_{ij}\in E_{t}\\ \infty \;,\;e_{ij}\notin E \end{array}\right.$$ (1)

Where $d_{ij}\in R$ is the distance between stations *i* and *j*, $v_{avg}\in R$ is the mean train speed, $t_{p}\in \;R$ is the time required to commute between lines within the same station, and $t_{t}\in R$ is the transfer time. The mean train speed is set to 28 km/h [73]. The waiting times in the NYC subway system can range from 3–5 min [74]. Thus, for our calculations, we set the time required to change platforms to 5 min, while the time required to transfer from one station to another is set to 8 min, i.e., 3 min for transferring and 5 min for waiting.

With the network *G* created and the different weights *W*_*ij*_ assigned to the corresponding edges, Dijkstra’s algorithm is used to calculate the shortest path between two stations, thus mimicking the mobility patterns of commuters [36]. Dijkstra’s computational cost is often set to *O* (|*E*| + |*V*|*log*|*V*|) [37, 38, 75].

### 2.5 PM_2.5_ exposure calculations

#### 2.5.1 Mean per capita exposure at the census block level

We assessed subway PM_2.5_ exposure by considering both the concentration of PM_2.5_ within the subway system and the duration of exposure. When individuals use the subway to commute to work, they typically spend time waiting on the station platform before boarding the train [74]. To calculate the exposure at the first station, we can multiply the on-platform PM_2.5_ concentration by the waiting time at the station. Subsequently, workers embark on the train and travel for several minutes, which could potentially be the longest part of their journey, depending on the distance covered. The exposure experienced on the train can be determined by multiplying the time spent on the train by the mean PM_2.5_ concentration measured onboard. During their commute, workers may also need to transfer trains. These transfers can involve waiting on the same platform as the initial train or walking to a different platform. To calculate the exposure during transfers, we consider the PM_2.5_ concentration at the transferring station and the duration of the transfer. It is possible for workers to have multiple transfers or train changes throughout their commute. We considered a constant 5-min waiting time for boarding and 3 mins for the exit station. The total exposure E can be expressed using the following notation:

$$E_{a,b}=t_{0}\;C_{i}+\sum_{\left(i,j\right)\in L}\left(d_{i,j}/v_{avg}\right)\;C_{i,j}\;+\sum_{p\in P}t_{p}C_{p}+\sum_{t\in TR}t_{t}C_{t}+t_{f}C_{f}$$ (2)

Where,

*E*_*a*,*b*_ = Exposure for moving from block ‘a’ to ‘b’

*t*_0_ = Waiting time at station *i*. This is the boarding station in block ‘a’

*C*_*i*_ = On-platform PM_2.5_ concentration on station *i*

*C*_*i*,*j*_ = On-train PM_2.5_ concentration between station *i* and station j

*L* = Set of subway line segments traveled for a trip

*P* = Set of transfers within same station for a trip

*TR* = Set of interstation transfers for a trip

*d*_*i*,*j*_ = Distance between station *i* and station *j*

*v*_*avg*_ = Mean train speed

*t*_*f*_ = Exiting time at last station f

**Fig 4:**
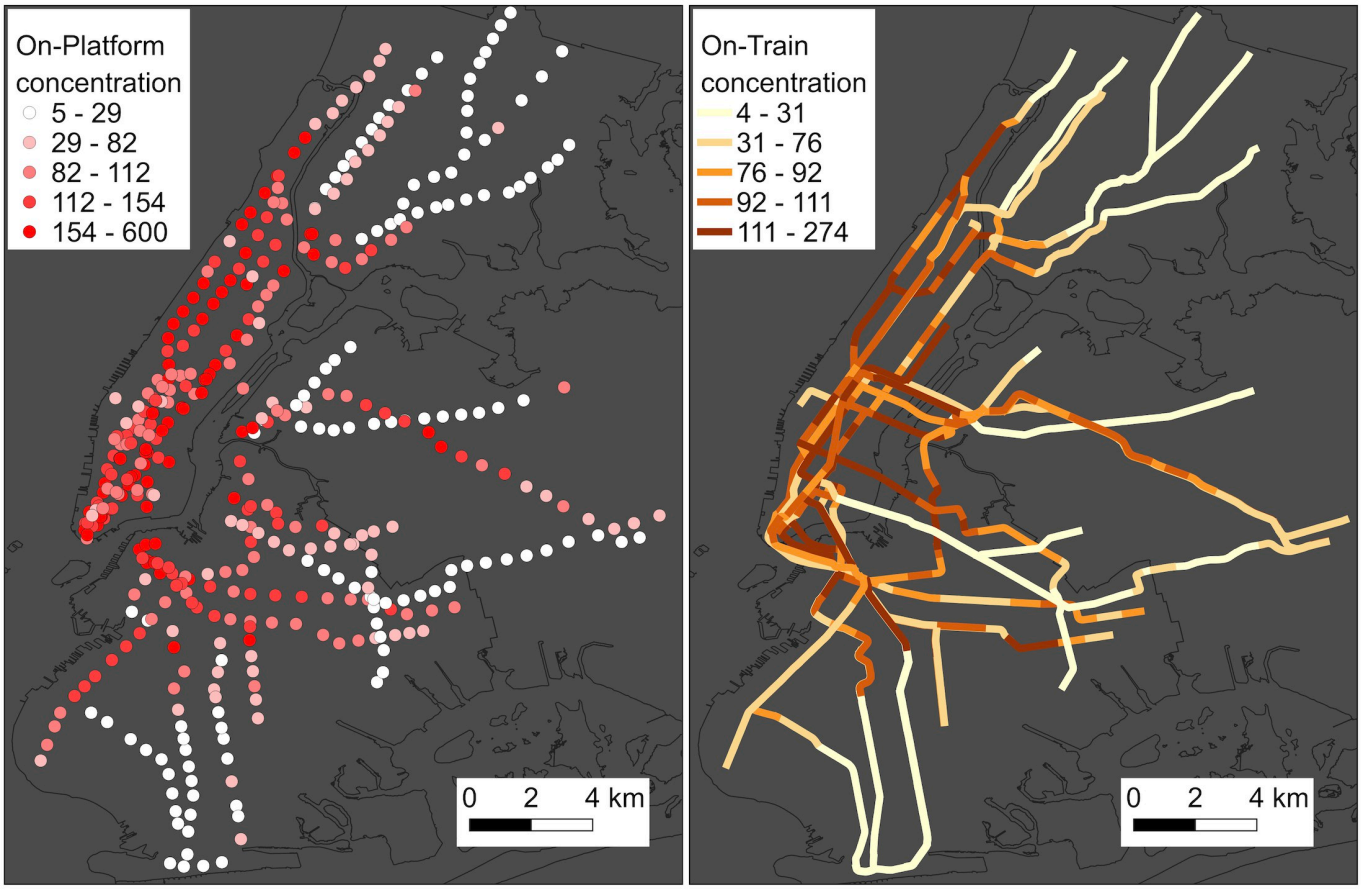
PM_2.5_ concentration in the subway system. (a) On-platform PM_2.5_ concentration and (b) on-train PM_2.5_ concentration.

In this study, we employed the network model described in Section 2.4 to identify the travel routes encompassing boarding, transfers, and destination stations, as well as the specific train lines used for commuting between blocks. Each train route corresponds to a measured PM_2.5_ concentration (as shown in Fig 4) and time, allowing us to calculate the overall exposure. The total travel time (TT) for a trip can be computed as:

$$\text{TT}=t_{0}+\;\sum_{\left(i,j\right)\in L}\left(d_{i,j}/v_{avg}\right)+\;\sum_{p\in P}t_{p}+\sum_{t\in TR}t_{t}$$ (3)

In order to determine the mean per capita PM_2.5_ exposure per census block, we used the weighted average technique. As illustrated in Fig 2, workers residing in a particular block may commute to various other blocks for work purposes. However, not all workers use public transportation for their daily commute. The percentage of workers using the public transit system to travel to their workplace at the census block level is demonstrated in Fig 2(d). The calculation of the mean per capita exposure for each census block can be expressed mathematically as Eq 4.

$${\text{PE}}_{\left(\text{a}\right)}=\frac{\sum_{i=1}^{n}W_{\left(a,b_{i}\right)}E_{\left(a,b_{i}\right)}}{\sum_{i=1}^{n}W_{\left(a,b_{i}\right)}}X\frac{\%\;subway_{\left(a\right)}}{100}X\;2$$ (4)

Where, PE_(a)_ = Per capita workers’ exposure for a round work-home subway trip

*W*_*a*,*bi*_ = total workers move from ‘a’ block to ‘*b*_*i*_’ block for work, (i = 1, 2, …, n).

*E*_*a*,*bi*_ = total PM_2.5_ exposure for moving to block ‘a’ to block ‘*b*_*i*_’

% *subway*_*a*_ = Percent worker in block ‘a’ uses subway to work

Here the *E*_(*a*,*bi*)_ is calculated by Eq 2, and *W*_*a*,*bi*_ comes from LEHD OD dataset described in section 2.1.

#### 2.5.2 Methods for correlation analysis

To understand the relation between the workers of different races as well as income groups and per capita mean exposure at census block level, we performed the bivariate correlation analysis with the Pearson correlation coefficient that can be determined with the following equation.

$$\rho \left(x,y\right)=\frac{Cov\left(x,y\right)}{\sigma_{x}\sigma_{y}}$$ (5)

Where, *ρ*(x,y) is the Pearson coefficient, Cov(x,y) is the covariance of x and y. σ_*x*_ and σ_*y*_ are the standard deviation of x and y, respectively. There is a total of 25,694 observations to calculate this correlation where each observation is a census block.

Additionally, we computed the mean PM_2.5_ exposure for different race and income groups. This is done with the equation below,

$$\text{ER}=\frac{\sum_{i=1}^{n}PE_{\left(i\right)}*R_{\left(i\right)}}{\sum_{i=1}^{n}R_{\left(i\right)}}$$ (6)

Here, ER is the mean exposure for one race or income group, PE_(i)_ is the per capita exposure for block i measured with Eq (4), and *R*_(*i*)_ is the population of the race group in block i.

## 3. Results and discussion

### 3.1 PM_2.5_ concentration in the NYC subway system

Results indicate on-platform underground mean and standard deviation concentration of 139 ± 25 μg/m^3^ versus aboveground values of 30 ± 11 μg/m^3^. On-train underground values were measured as 99 ± 21 μg/m^3^ versus aboveground 25 ± 18 μg/m^3^. These readings contrast with the 24-hr PM_2.5_ exposure guideline value of 15 μg/m^3^ set by the World Health Organization (WHO), which means 24-hour mean exposures should not exceed 15 μg/m^3^ more than 3 to 4 days per year [76]. It should be noted that a subway ride is not an entire day; hence, results cannot be directly contrasted with WHO’s mean daily guideline. However, it does provide some context or baseline to understand the extent of exposure during a daily commute. Fig 4(a) shows the on-platform PM_2.5_ concentration for 429 stations from 19 subway lines. Fig 4(b) shows the on-train concentration of 19 subway lines. We have created an interactive platform that can be used to calculate personal exposure for any origin and destination within NYC. This can be accessed at https://bit.ly/47kTcDh.

### 3.2 Quantifying personal exposure with the network model

**Fig 5:**
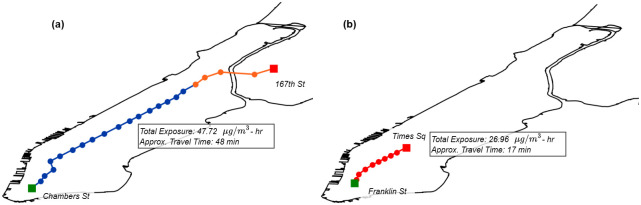
Personal subway PM_2.5_ exposure. (a) Exposure for a sample trip from the Bronx to Downtown Manhattan (b) Exposure for a sample trip from Midtown to Downtown Manhattan.

We have implemented the network-based model above on a server with the measured air pollution concentrations. In this way, users can select two locations on the map (origin and destination) and get the exposure level for their subway trip. The output consists of the approximate travel time and total exposure associated with that subway trip. Fig 5 shows examples of two different trips.

In the example of the trip from the Bronx to Downtown Manhattan, Fig 5 (a), a line change is needed, resulting in a travel time of approximately 48 min for a total exposure of 47.72 μg/m^3^-hr. On the other hand, the trip from Midtown to Downtown Manhattan, Fig 5(b), shows a case where no transfer is needed, where the travel time is short (17 min), resulting in a total exposure of 26.96 μg/m^3^-hr.

### 3.3 Quantifying per capita exposure at the census block level

**Fig 6:**
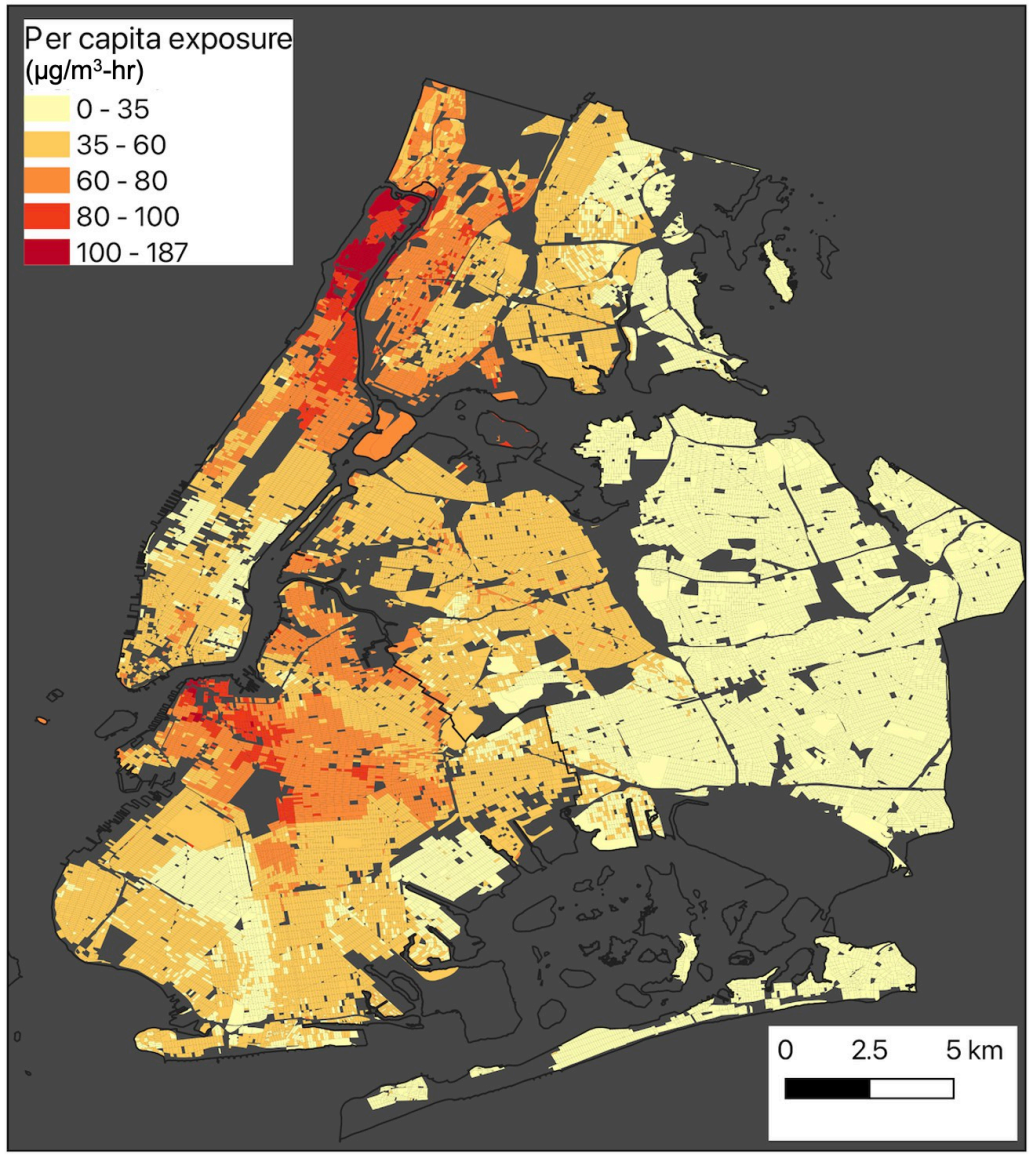
Mean per capita exposure at census block level.

In addition to personal exposure, we also computed the per-capita census block level exposure to understand the scenario at the community level. In Fig 6, a detailed representation is provided, illustrating the per capita exposure to PM_2.5_ at the census block level. The exposure metric used in this context is expressed in units of micrograms per cubic meter per hour (μg/m^3^-hr). To provide a comprehensive understanding of per capita exposure, let us examine the northwestern part of Queens as an example. In this area, the per capita exposure to subway air pollution appears to be lower compared to other areas (Fig 6). However, workers residing in these outskirts and utilizing the subway system may encounter longer commuting times due to their distance from the job center, such as midtown. Consequently, these workers may experience high subway PM_2.5_ exposure. It should be noted that the majority of workers residing in these areas do not rely on the subway for their daily commute (Fig 2d). Therefore, while those who use the subway may face elevated exposure, the overall mean per capita exposure remains lower due to the significant number of workers who do not utilize the subway system. So, it is important to note that per capita exposure is a metric to compare exposure at the community level and is not suitable for measuring individual or personal exposure. For accurately assessing personal exposure during a subway trip, we recommend employing the network model described in section 3.2.

### 3.4 Inequality in subway PM_2.5_ exposure

**Fig 7:**
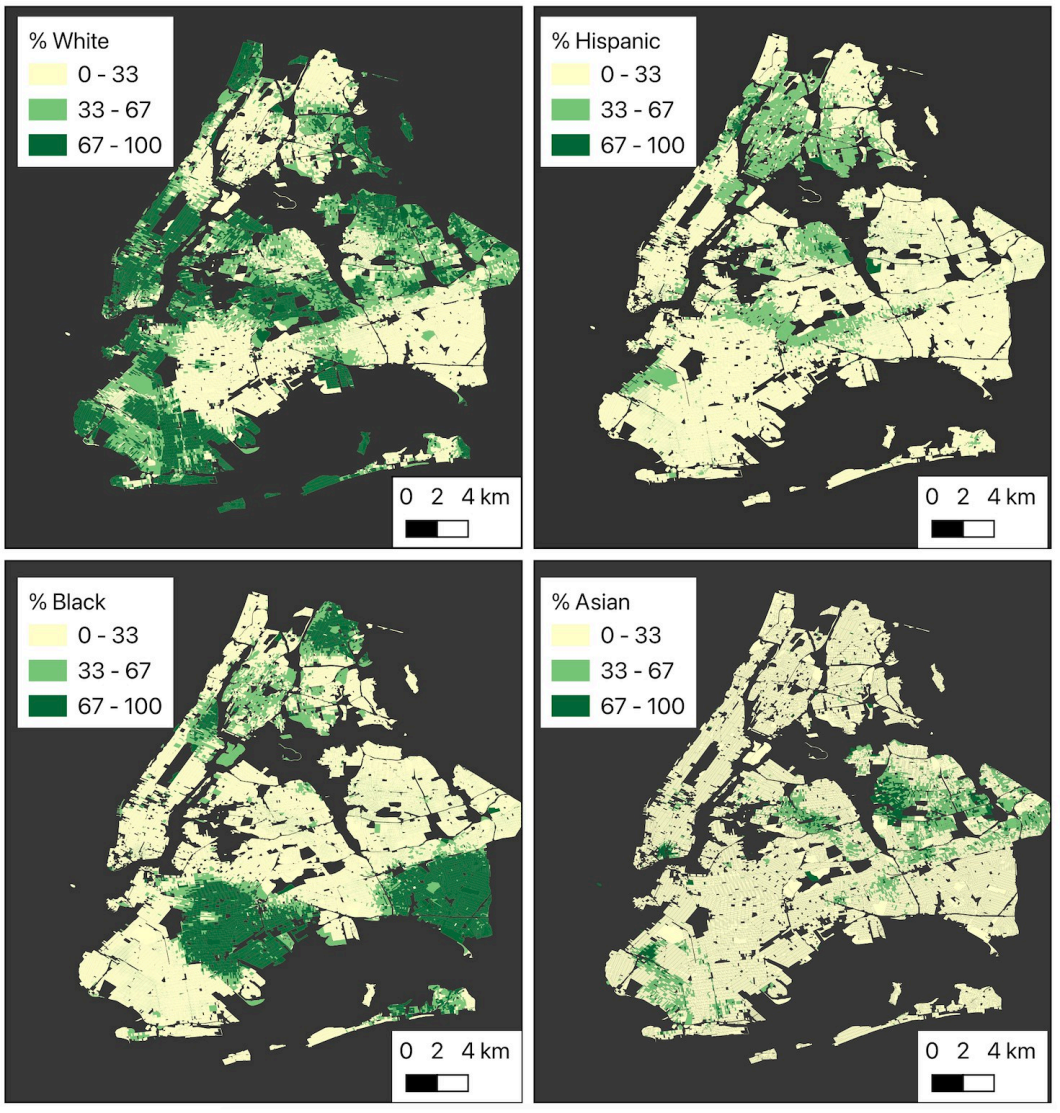
Spatial distribution of different racial groups in the city at census block level. (a) percent of White workers, (b) percent of Hispanic workers, (c) percent of Black workers, (d) percent of Asian workers.

In this section, we have explored the socioeconomic dimensions of subway PM_2.5_ exposure by focusing on economic and racial disparities. The downtown and midtown Manhattan areas are mostly inhabited by White workers. These areas serve as the city’s central business districts and major job hubs. These residents live close to their workplace, so they have shorter commuting distances and a lower possibility of extensive subway PM_2.5_ exposure. Conversely, upper Manhattan and the Bronx offer comparatively fewer job opportunities and have a higher proportion of Black and Hispanic workers. Southwest Queens and Brooklyn also have a sizable population of Black workers. Due to the limited job opportunities in these areas, many workers commute longer distances to their workplaces. Asian workers, although constituting only around 11% of the total workforce in the city, are concentrated in specific areas, including south-downtown Manhattan (Chinatown). West Brooklyn and East Queens also have significant Asian populations. Fig 7 shows the distribution of workers of different races.

The Pearson correlation coefficients between the percentages of White and Asian workers and subway PM_2.5_ exposure are -0.22 and -0.23, respectively. These are statistically significant, but weak negative correlation coefficients indicate that areas with a higher percentage of White and Asian workers may have slightly lower levels of subway-driven PM_2.5_ exposure. Conversely, the correlation coefficients between the percentages of Hispanic and Black workers and subway exposure are +0.38 and +0.18, respectively. These weak but statistically significant ((p-value < 0.05) positive correlation coefficients suggest that areas with a higher percentage of Hispanic and Black workers may be associated with high subway PM_2.5_ exposure.

**Fig 8:**
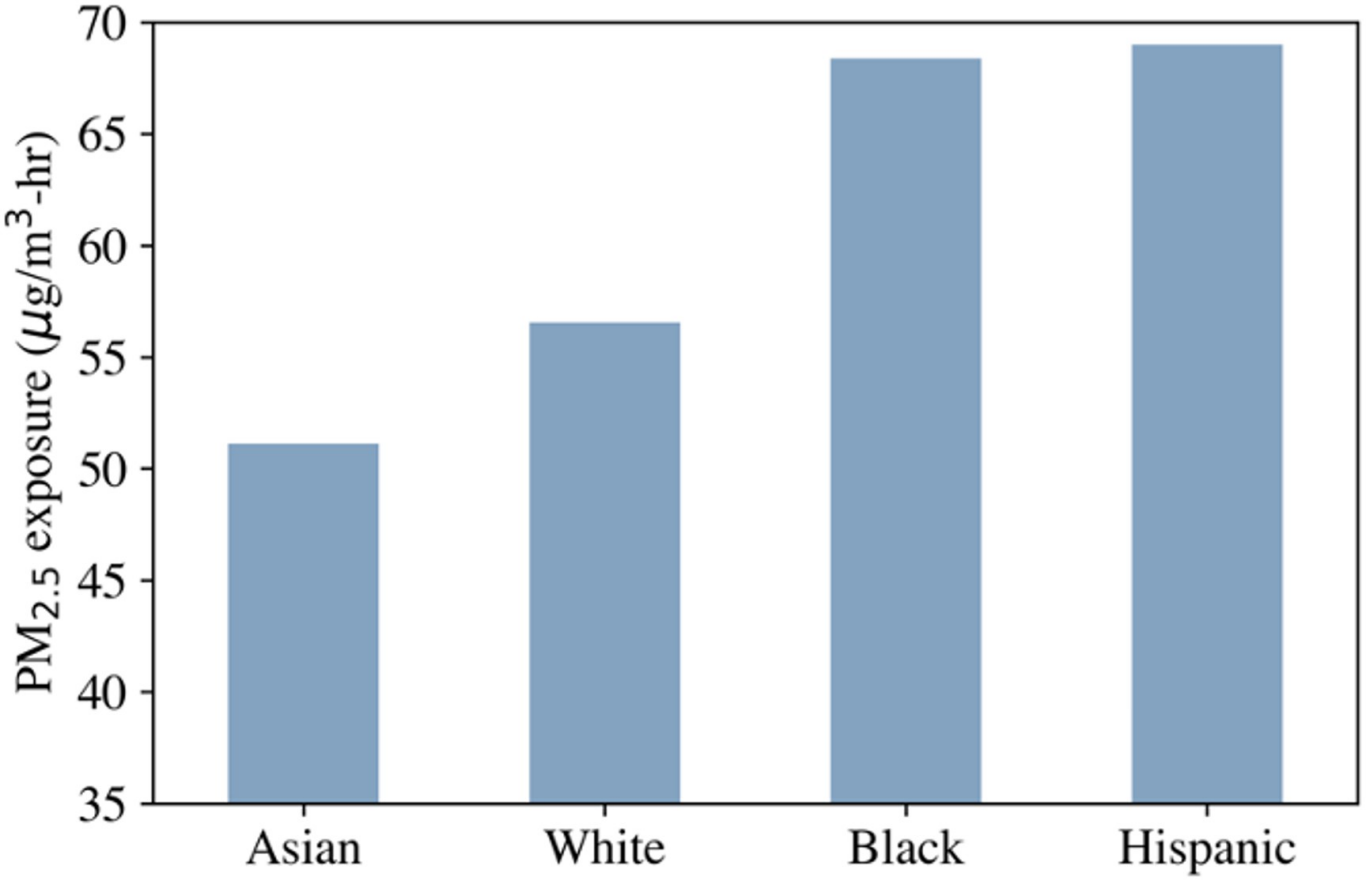
Mean exposure for different race groups.

We also quantified the mean PM_2.5_ exposure for different race groups. Fig 8 shows the mean per capita exposure for different race groups. The numbers indicate that Asian and White workers are exposed to lower concentrations of subway PM_2.5_ compared to Black and Hispanic workers. The mean exposure for Asian workers is 51 μg/m^3^-hr, while White workers have a mean exposure of 56 μg/m^3^-hr. However, both Black and Hispanic workers experience a mean exposure of 69 μg/m^3^-hr. This means that Black and Hispanic workers are exposed to 35% higher subway PM_2.5_ exposure compared to Asian workers.

Moving on to the analysis of the exposure of different income groups, the correlation analysis reveals a weak positive Pearson correlation coefficient of +0.36 between subway PM_2.5_ exposure and the size of the population below the poverty threshold at the census block level, a statistically significant positive relation. Looking at it from a different angle, a statistically significant negative Pearson correlation coefficient of -0.23 was found between subway exposure and median family income. This weak negative correlation indicates that higher income levels have a chance to associate with lower levels of subway PM_2.5_ exposure. A note on the income data: the available poverty and income data from the U.S. Census Bureau is reported at the census tract level. In order to conduct our correlative analysis, we spatially disaggregated the census tract data to the census block level, allowing us to examine the relationship between subway exposure and socioeconomic factors at a more granular level.

**Fig 9:**
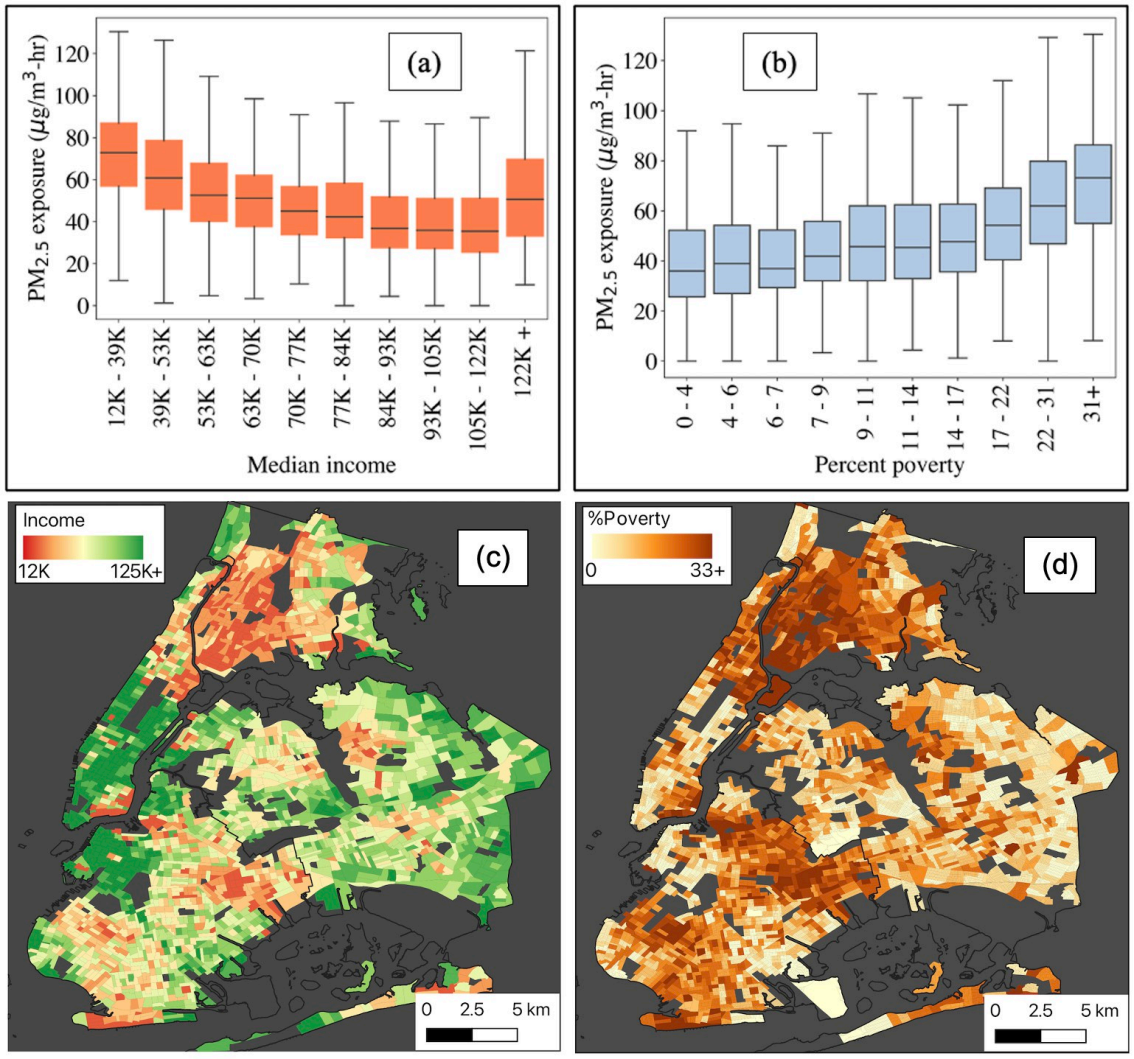
Mean exposure in different income levels. (left) Box plot shows the exposure level in different income groups, (right) relation of exposure level with poverty.

The mean per capita subway PM_2.5_ exposure within a community can be influenced by the percentage of workers relying on the subway system. Higher dependence on the subway corresponds to a higher mean per capita exposure to PM_2.5_. Fig 9(a) shows the relationship between exposure and income and exposure and poverty in box plots. Here, first we divide the census block level income and poverty data into deciles (divide data into ten equal parts), and the distribution of exposure in each decile is shown in a box in the plot. The horizontal line inside the box represents the median exposure value for each group and the whiskers extending from the box represent the minimum and maximum values of the data, excluding any outliers. From the figure, it is evident that the lowest income group in the city, with a median family income between 12,000 and 39,000, experiences the highest levels of subway PM_2.5_ exposure. As the income level increases, there is a gradual decline in exposure levels until reaching the highest income groups. Fig 9(b) reveals a clear relationship between the percentage of individuals living below the poverty line and subway PM_2.5_ exposure. Areas with a low percentage of people below the poverty line (less than 4%) exhibit the lowest levels of subway PM_2.5_ exposure. However, as the percentage of people living below the poverty line increases, the exposure levels also gradually rise. Fig 9(c) shows the spatial distribution of the median household income in the city. Fig 9(d) shows the spatial distribution of the percentage of poverty in the city.

To some extent, the discrepancy in subway usage between income groups can be attributed to high-income workers having access to alternative transportation options, such as private vehicles or carpool services. Fig 2(a) highlights that many workers residing in downtown and midtown Manhattan have the convenience of working in the same block where they live, eliminating the need for subway usage. This stands in contrast to workers residing in other parts of the city who must undertake longer commutes to reach their workplaces. However, there are some notable anomalies; for example, one of the highest household income groups experiences high levels of exposure when using the subway. This is because these census blocks are heavily populated with high-income workers and use highly polluted subway stations, as shown in Fig 4.

### Limitations

While the reported high concentration of PM_2.5_ is alarming, a health outcomes analysis of that exposure is complex since health outcomes guidelines (by US EPA and WHO) are largely based on particulate matter from fossil fuel combustion. Health impact analysis of inhalation of iron-based particles is needed to contextualize the results presented here.

This study relied on data related to workers’ mobility for their daily commutes from home to work to estimate community-level subway exposure. However, achieving more precise results would have been feasible if it had been possible to include all types of trips, not only home-to-job commutes, and considered all residents, not just the working population. Such enhancement could be achieved through multiagent simulation modeling and is an area to be explored in future research.

## Conclusion

Through this study, it was possible to examine the population exposure to PM_2.5_ in the NYC subway system. This was accomplished using a network model that incorporated the commuter’s origin-destination data, as well as measured fine particulate matter concentrations throughout the system (platforms and cars), resulting in the mean per capita PM_2.5_ exposure of 3.1 million working commuters across 34,169 census blocks in NYC.

The study revealed the presence of exceptionally high concentrations of iron rich fine PM_2.5_, where mean concentrations on platforms and train cars were measured to be 10 times and 7 times the 24-hr guideline set by WHO, respectively. We quantified the disparities in the subway PM_2.5_ exposure across different racial and income groups. Results indicate weak positive correlation between elevated subway PM_2.5_ exposure and racial minority as well as economically disadvantaged groups. Results show statistically significant weak positive correlation between high subway PM_2.5_ exposure and lower-income working communities. We also found that Black and Hispanic workers experience 35% and 23% higher PM_2.5_ exposure, respectively, compared to Asian and white workers.

One of the primary contributors to this disparity is the difference in commuting patterns observed among subway users. Different workers have distinct origins and destinations, resulting in variations in the stations they pass through during their home-job subway trips. As a consequence, exposure to PM_2.5_ can significantly differ based on the specific route taken and the stations encountered along the way.

Additionally, Individuals with longer commutes or those who frequently transfer between subway lines may spend more time in the subway environment, potentially leading to increased exposure to air pollution. We found that certain subway stations exhibit higher concentrations of PM_2.5_ than others. Consequently, commuters who spend time at these stations for transferring or boarding are subject to increased exposure.

We also found that the on-train concentration of PM_2.5_ increases in certain subway lines when passing through specific underground tunnels. This implies that commuters whose subway routes include these lines and tracks may be exposed to higher concentrations within the train cabins.

The mean per capita subway PM_2.5_ exposure within a community can be influenced by the proportion of workers who rely on the subway system for their daily commute. A higher dependence on the subway is associated with a higher mean per capita exposure to PM_2.5_. We observed that workers residing in low-income communities tend to have a greater reliance on subways compared to workers in more affluent communities. Therefore, socioeconomic factors play a role in shaping the transportation choices of individuals and subsequently impact their exposure to subway driven PM_2.5_.
